# Editorial: Technological evolution in minimally-invasive radical prostatectomy. From laparoscopic to single-port robotically assisted

**DOI:** 10.3389/fsurg.2026.1878690

**Published:** 2026-06-01

**Authors:** Gernot Ortner, Theodoros Tokas

**Affiliations:** 1 LKH Hall in Tirol, Hall in Tirol, Austria; 2 University Hospital of Heraklion, Heraklion, Greece

**Keywords:** artificial intelligence (AI), future perspective and strategies, interdisciplinary, minimally-invasive surgery, robot-assisted prostatectomy, single port, telesurgery

Over the past two decades, minimally invasive surgery has substantially reshaped the management of localized prostate cancer. The transition from conventional laparoscopic radical prostatectomy to robot-assisted multiport techniques, and more recently to single-port approaches, reflects a continuous effort to reduce surgical morbidity while preserving oncological efficacy and improving functional outcomes. This Research Topic was conceived to capture key elements of this development and to place recent advances within a broader interdisciplinary context.

The evolution from laparoscopy to robotic-assisted surgery represents not merely a technical refinement but a shift towards greater precision and standardization. As outlined by Nick et al., contemporary prostatectomy increasingly integrates enhanced visualization, improved instrument articulation, and adjunctive technologies such as image guidance and data-driven support systems. These developments contribute to reproducibility and facilitate more tailored surgical approaches, while also indicating a gradual transition towards more structured and technology-supported workflows.

At the same time, the definition of surgical success has broadened. While oncological control remains the primary objective, functional outcomes—particularly urinary continence and sexual function—have become increasingly important. The contributions within this Research Topic highlight how this shift is closely linked to a more detailed understanding of pelvic anatomy and to the refinement of surgical technique. The work by Li et al. underlines the relevance of the membranous urethra, whereas Rassweiler et al. emphasize the importance of preserving periurethral supporting structures and performing appropriate reconstructive steps. These approaches are indicative of a broader development towards anatomy-based surgery, where detailed structural knowledge informs intraoperative decision-making and contributes to improved functional recovery.

Advances in preoperative imaging further support this trend. Multiparametric magnetic resonance imaging has become an integral component of preoperative assessment and surgical planning. As demonstrated by Galante Romo et al., imaging findings increasingly influence surgical strategy and expectations regarding both oncological and functional outcomes. At the same time, the limitations of current imaging techniques remain evident, indicating that imaging should be interpreted in conjunction with clinical judgment rather than as a standalone determinant.

In parallel, the physiological implications of minimally invasive techniques have led to a growing awareness of perioperative factors. The study by Liu et al. illustrates how aspects such as patient positioning and pneumoperitoneum may affect intraoperative parameters, including intraocular pressure. These observations highlight the importance of close collaboration between surgical and anaesthesiological teams and underscore that optimizing outcomes requires a comprehensive perioperative approach.

Taken together, the contributions in this Research Topic suggest that the ongoing development of minimally invasive radical prostatectomy is characterized by the interaction of multiple domains. Surgical technique, imaging, perioperative management, and technological innovation are increasingly interdependent. Progress in one area is closely linked to developments in others, reinforcing the importance of interdisciplinary collaboration.

Looking ahead, further changes may arise from the introduction of new robotic platforms and the increasing diversification of the surgical technology market. Greater competition may improve accessibility and potentially lower costs, thereby facilitating wider adoption. In this context, telesurgical concepts and teleproctoring may become increasingly relevant. Remote guidance and structured training pathways could help disseminate complex surgical techniques beyond specialized centers and may offer opportunities to expand access to advanced procedures in lower-resource settings.

Nonetheless, such developments require careful evaluation. Issues related to training, quality assurance, infrastructure, and regulatory frameworks will need to be addressed to ensure that broader accessibility does not compromise patient safety or outcomes. The integration of new technologies into clinical practice should therefore be accompanied by rigorous assessment and ongoing multidisciplinary exchange.

In summary, this Research Topic reflects the dynamic development of minimally invasive radical prostatectomy and illustrates how technological innovation, combined with an improved understanding of anatomy and physiology, continues to influence surgical practice. The contributions highlight the importance of a balanced approach that aligns technical progress with clinical judgment and interdisciplinary collaboration. Future developments will likely depend not only on further technological advances but also on the ability to integrate these innovations into safe, effective, and accessible patient care.

